# Identifying the Hernial Orifice in Superior Lumbar Hernia Repair by Utilizing Anatomical Landmarks on Preoperative CT

**DOI:** 10.7759/cureus.39154

**Published:** 2023-05-17

**Authors:** Ryujiro Akaishi, Sho Fujiwara, Momoka Ando, Hiroshi Suzuki, Toru Hoshida

**Affiliations:** 1 Surgery, Iwate Prefectural Ofunato Hospital, Ofunato, JPN

**Keywords:** anatomical landmarks, ct scan, hernial orifice, lumbar hernia repair, lumbar hernia

## Abstract

Superior lumbar hernias are extremely rare, and surgical repair is essential for their treatment. However, the direct observation of the hernial orifice is frequently difficult because the hernia disappears in prone or lateral positions, which is an issue when using the open approach. Therefore, using anatomical landmarks to detect the hernial orifice on preoperative CT scans may be useful for correct identification and visualization. Here, we report two cases of superior lumbar hernias successfully treated using the abovementioned method.

## Introduction

Lumbar hernias (LHs) are extremely rare hernias where abdominal contents protrude through a defect in the dorsal abdominal wall, of which approximately 300 cases have been reported to date [1]. Superior lumbar hernias (SLHs) are herniations of abdominal contents through the superior lumbar triangle, bordered by the 12th rib, internal oblique muscle, and erector spinae or quadratus lumborum muscles [2]. According to a previous study, 30.8% of patients with LHs had incarcerated hernias, and one case of strangulated bowel obstruction has been reported [3,4].

Surgical repair is the most effective treatment for LHs [5]. However, the surgical approach presents technical difficulties in visualizing the hernial orifice because of its location, inadequate fascia, and weakness of the surrounding tissue [3]. LH is a rare condition that is frequently misdiagnosed; therefore, surgical strategies are yet to be established.

In this case report, we describe two cases of SLHs that underwent open-approach repair using anatomical landmarks to identify the hernial orifice with the assistance of preoperative imaging. Furthermore, we aimed to help develop surgical strategies to overcome the difficulties associated with this type of hernia.

## Case presentation

Case one

**Figure 1:**
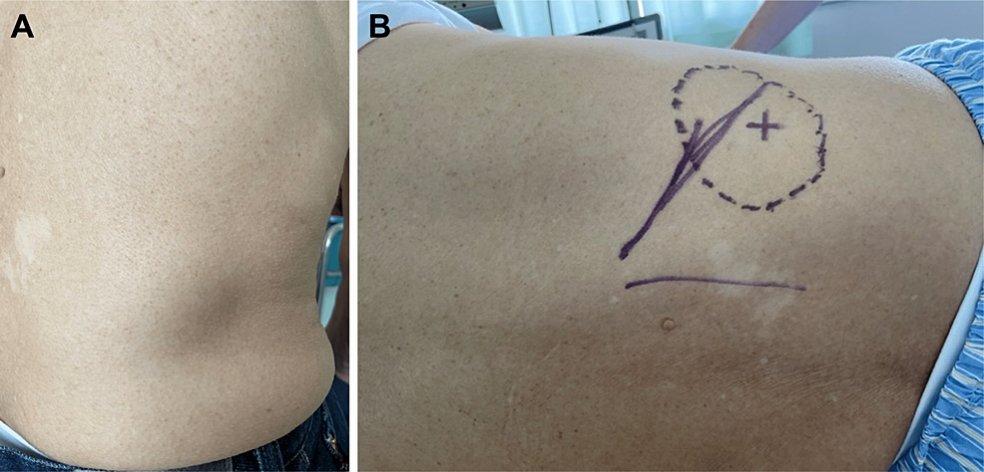
Patient in the sitting and left lateral positions. A: The hernia protruding in the sitting position. B: The hernia disappearing in the left lateral position.

An 80-year-old male patient with a history of myocardial infarction, idiopathic thrombocytopenic purpura, and laparoscopic cholecystectomy was referred to our hospital. He reported the presence of a protruding mass in the right lumbar area for one week without any preceding episode of trauma. On physical examination, a soft mass measuring 40 × 40 mm and protruding in the standing and sitting positions was observed at the right costovertebral angle. However, the protrusion disappeared in the prone and lateral positions (Figure 1).

**Figure 2:**
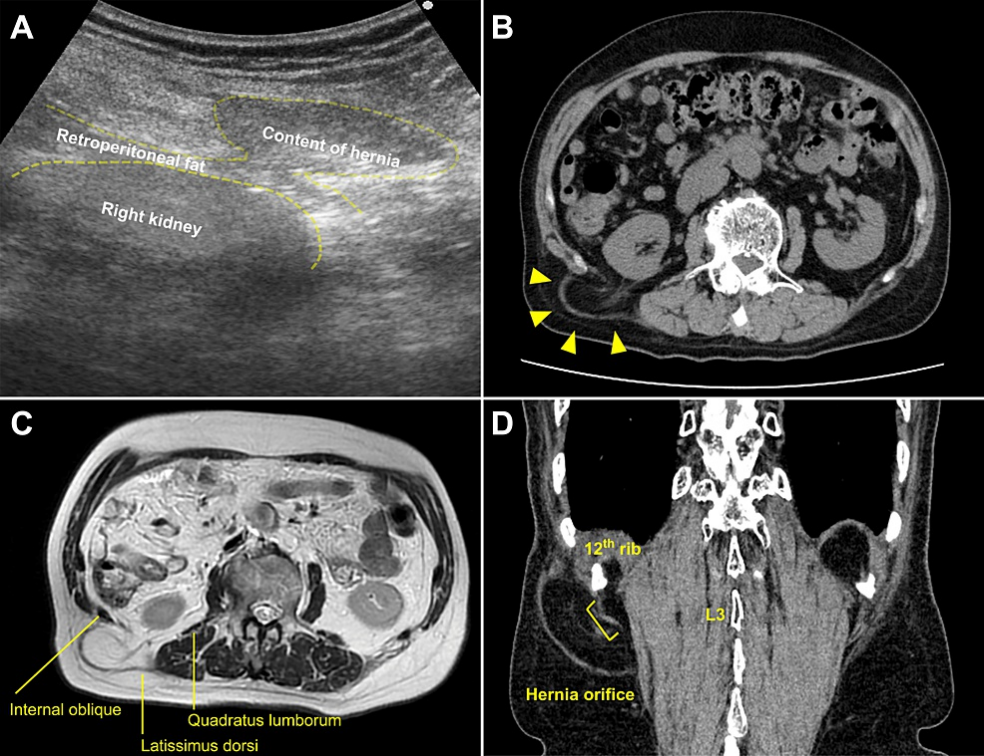
Preoperative imaging. A: Ultrasonography of the superior lumbar hernia. B: CT scan: Retroperitoneal fat directly protruding from the hernial orifice. C: MRI: The hernial orifice in relation to the 12th rib, internal oblique muscle, and quadratus lumborum muscle. D: The hernial orifice is oriented in relation to the right 12th rib and processus spinosus of the third lumbar vertebrae.

**Figure 3:**
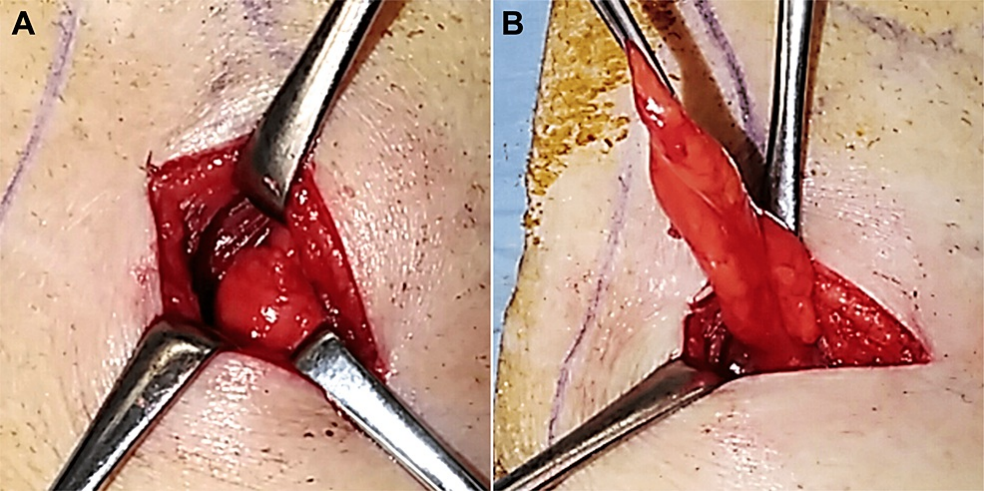
Surgical findings. A: The hernial orifice. B: Retroperitoneal fat without a hernia sac.

Ultrasonography revealed a retroperitoneal adipose tissue mass. CT and MRI showed a right SLH. The patient underwent a hernioplasty for the right SLH. The hernial orifice, oriented in relation to the right 12th rib and processus spinosus of the third lumbar vertebrae, was preoperatively marked according to the CT scan for correct detection (Figure 2). The hernioplasty was performed in the prone position with a 4-cm skin incision. A 1.2-cm hernial orifice with protruding retroperitoneal fat without a hernia sac was identified beneath the fragile latissimus dorsi muscle. The herniated tissue was dissected from the hernial orifice and returned to the retroperitoneal space. A 7.6-cm diameter Bard® Modified Kugel® Hernia Patch Direct sub-lay mesh was placed into the retroperitoneal space, and the strap was fixed to the transversalis fascia with a 2-0 braided absorbable suture. A Bard® onlay mesh was cut in a 5.0-cm diameter and then placed and stitched onto the latissimus dorsi (Figure 3). The patient was discharged on postoperative day three without complications. No recurrence or late-onset complications occurred three months postoperatively.

Case two

**Figure 4:**
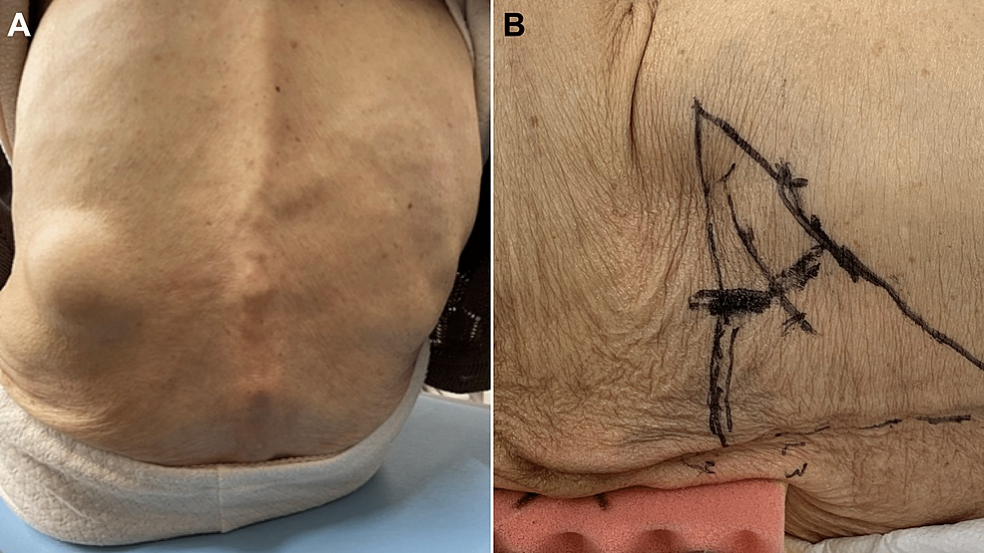
Patient in the sitting and right lateral positions. A: The hernia protruding in the sitting position. B: The hernia disappearing in the right lateral position.

**Figure 5:**
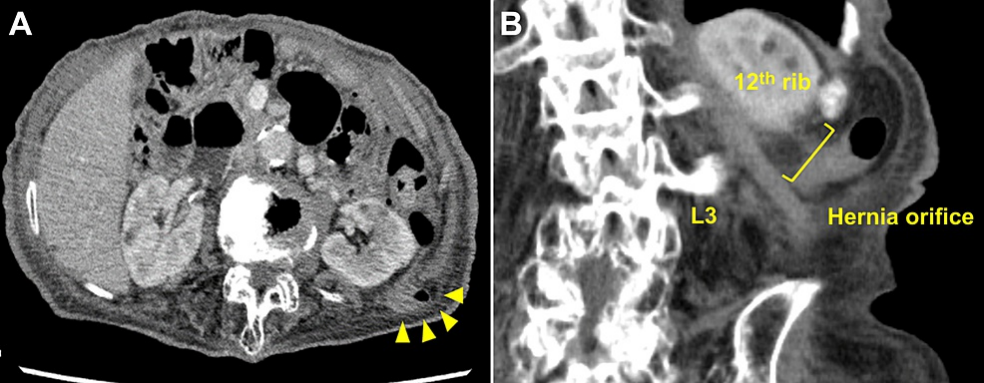
Preoperative imaging. A: Small bowel protruding into the hernia sac. B: The hernial orifice is oriented with regard to the left 12th rib and left processus transversus of the third lumbar vertebrae.

A 90-year-old female patient with a medical history of cerebral infarction and a hiatal hernia, although generally self-reliant, presented with a protruding mass in the left lumbar region. Physical examination revealed a round and soft protruding mass beneath the 12th rib of the left upper flank area. The mass easily reduced and protruded while sitting. However, the mass disappeared in the prone and lateral positions (Figure 4). Ultrasonography revealed low-echoic content within the hernial sac, which was suspected to be intestines. CT scan revealed small intestines protruding toward the left lumbar subcutaneous fat, which was diagnosed as a left SLH (Figure 5).

**Figure 6:**
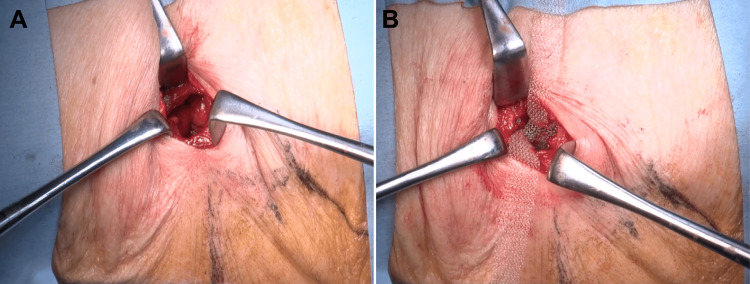
Surgical findings. A: The hernial orifice after dissection and ligation of the hernia sac. B: Sub-lay mesh placed into the retroperitoneal space.

Considering the potential risk of strangulation, the patient underwent a hernioplasty. The hernial orifice, oriented in relation to the left 12th rib and left processus transversus of the third lumbar vertebrae, was marked preoperatively according to the CT scan for correct detection. An open hernioplasty was performed with the patient in the right lateral position via a 4-cm skin incision. A 2.5-cm hernial orifice was identified, and the hernial sac was dissected and ligated. A 10.2-cm diameter Bard® Modified Kugel® Hernia Patch Direct sub-lay mesh was placed into the retroperitoneal space, and the strap was fixed to the hernial orifice with a 3-0 braided absorbable suture (Figure 6). The patient was discharged on postoperative day three without complications.

## Discussion

A significant clinical issue in repairing SLHs is correctly identifying and visualizing the hernial orifice. Generally, the patient is positioned in the prone or lateral position during LH repair [6]. In cases with a small hernial orifice, such as those mentioned in this case report, these positions obscure the hernia and make it difficult to correctly identify the orifice. CT scan is essential in defining hernia contents accurately [4]. However, ultrasonography and MRI may not diagnose LH without an obvious bulge in the flank region [7]. Preoperative CT scan images were used to mark the hernial orifice in relation to anatomical landmarks, such as the 12th rib and processus spinosus, and the processus transversus of the lumbar vertebrae was useful for deciding the skin incision line over the hernial orifice.

Laparoscopic hernioplasties using a mesh that enables easy visualization of the hernial orifice have recently been reported [8]. However, both our patients were taking antiplatelet medications or anticoagulants, and an open approach was selected considering minimal skin incision and retroperitoneal dissection. Furthermore, tension-free mesh hernioplasty appears more effective than conventional herniorrhaphy, particularly in patients with muscle atrophy [1]. Onlay, sub-lay, and sandwich (onlay + sub-lay) techniques have been reported as open tension‐free repair. However, onlay repair was reported with the highest recurrence rate among the open tension-free repair methods and is usually applied as a supplemental procedure [9]. A small incision directly above the hernial orifice enabled minimal dissection and facilitated tension-free mesh hernioplasty without difficulty in the abovementioned cases.

## Conclusions

Tension-free mesh hernioplasty using an open approach based on radiographic hernial orifice identification is a simple and feasible technique for treating SLH. SLH is an extremely rare condition. Therefore, our report may be helpful to surgeons treating SLHs.

Furthermore, the small incision directly above the hernial orifice is effective, with minimal tissue dissection necessary, and ensures the exact placement of the mesh. Nevertheless, further studies are required to validate the efficacy and superiority of these findings.
